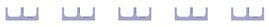# Simple Discriminatory Methodology for Wear Analysis of Cutting Tools: Impact on Work Piece Surface Morphology in Case of Differently Milled Kinetics Steel H13

**DOI:** 10.3390/ma13010215

**Published:** 2020-01-04

**Authors:** Teresa Prado, Alejandro Pereira, Maria Fenollera, Thomas G. Mathia

**Affiliations:** 1 Manufacturing Engineering Group (GEF) EEI Campus Lagoas, University of Vigo, 36310 Vigo, Spain; apereira@uvigo.es (A.P.); mfenollera@uvigo.es (M.F.); 2 Laboratoire de Tribologie et Dynamique des Systèmes LTDS, École Centrale de Lyon, 69134 Ecully CEDEX, France; Thomas.Mathia@ec-lyon.fr

**Keywords:** cutting tools, wear kinetics, surface morphology

## Abstract

Recently, there is growing interest in optimisation of finishing process thanks to the technologies to follow online the wear of cutting tools. In the present paper, one of the cheapest and simplest non-contact methodologies is described in detail and investigated with robustness evaluation. To simulate the finishing operation of a die, in this study, two cavities were designed in AISI H13 steel. Different inserts corresponding to PVD-(Ti,Al)N coated cemented carbide tool were tested. The described methodology is easy to be applied in manufacturing cutting machining with the opportunity to be implemented on machining processes to follow reasonably wear process of cutting tools.

## 1. Introduction

In any mass manufacturing process the constant quality production is crucial. The respect of functionality of machined surfaces is a fundamental restriction for manufacturers.

During the last 20 years a great effort has been done in selection of functional parameters for tribological application as sliding, breaking, and adhesion, or optical such as brightness, etc. [1]. However, there are no simple relationships between these functional parameters and manufacturing process [2]. The metrology of surface morphology of produced surface is generally done in frame of post-production control process essentially for simplicity and economic reasons. Different techniques are currently used [3]. If the acquisition strategy requires the knowledge of transfer function of metrological device, there are similarly needs of software algorithms for data treatments. The most of existing data treatments software for surface morphology analysis are offering automatic parameters calculations [4]. Apart of that few restrictions, the post-machining roughness control cannot be sufficient in order to prevent nonconformity in production and therefore a great number of defective pieces. Therefore zero-defect production becomes the dream. Optimisation of mass production requires not only the perfect master of high precision machine tool kinematics [5,6,7] but also strict knowledge of tools definition and its wear kinetics [8,9,10,11]. A detailed critical bibliography is described deeply in [12].

**Figure 1:**
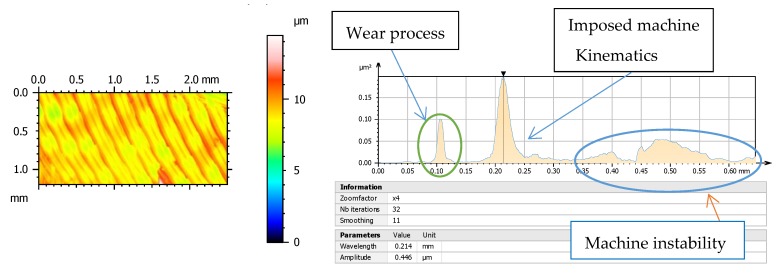
Power spectral density (PSD) function offers the possibility of quantitative identification of certain motifs, the periodicity, and their orientation from 3D analysis.

Morphology of machined surfaces contains a great number of information concerning the process conditions as tools, work-piece kinematics, and also its evolution during machining process. Therefore wear of cutting edges can be stated as can be easily observed from the power spectral density function (PSD) in Figure 1.

Spectral analysis enables us to determine the periodicity and orientation of certain motifs that exist in addition to roughness, by showing the frequencies (or wavelengths) found in the spectrum. This spectrum is obtained using the Fourier transform. The vertical axis displays the morphology amplitude to a power of 2. The horizontal axis is graduated in wavelengths. The values related to the peaks in Figure 1, analysed in a specimen of this work, show the dominant wavelengths indicating morphological compounds reflecting wear process and machine impact on manufacturing process. Along the same lines, the team of Krolczyk uses PSD to characterize turning regarding applied cooling methods and they state that PSD in analysed surfaces is a method of quantitative comparison surface quality by means of harmonic components [13].

One of the factors most influential on work piece surface morphology is the wear of the cutting tool. Research in the area of prediction and monitoring of tool wear is very extensive. Shiraishi et al. classify wear measurement technologies into two types: direct and indirect methods [14]. On one hand, direct methods are based on cutting edge geometry changes by using sensors such as optical, electrical or radioactive [15]. Its application is simple and reliable high due to the seizing of geometry in real time, but its automation is difficult under unfavourable environmental conditions because of refrigerant use or chip formation. On the other hand, indirect methods use the effects caused by tool wear. They are based on calibration procedures. This involves process parameters measurement that correlate with wear (cutting force, vibration, sound, acoustic emission, temperature, energy consumption, or surface roughness) [16]. They are easy-to-apply methods, but their reliability is limited regarding direct methods [17].

A reliable tool condition monitoring system should enable optimal tool lifecycle utilization. As Botsaris and Tsanakas argue, indirect methods seem more effective at controlling progressive failures, i.e., for wear prediction, whereas direct methods are generally used for diagnosing unpredictable failures, such as tool breakage [18].

This research focuses on finding methods of monitoring and measuring tool wear in machining processes. Wear analysis is approached from an optical point of view and this analysis is linked to the surface quality of parts, measuring the topography of the surfaces both in the workpiece and in the cutting tool edge. For the present article, milling process of a die offering various contact mechanics situations of cutting edge and work-piece with complex kinematics is discussed.

The first optical methods were principally used offline by adapted microscopes to allow digital processing of cutting edge tool. The improvement of productivity during production in the industry demands to introduce these methods in the real-time inspection of the tool and its geometric parameters. Therefore, subsequent studies focused on online monitoring systems of the tool wear [19]; most of them are related the influence of tool wear in surface quality as the machined surface is a replica of the cutting edge state [20]. 

Under this premise the present research analyses topography of the parts and the cutting edge tool in order to correlate them with the state of the tool. This relationship would allow knowledge of the moment to change tool so as to obtain a specific surface quality, thereby optimising the manufacturing process as well as its cost. In this work an original direct optimal methodology is introduced and linked to an indirect methodology based on the analysis of machined surfaces’ morphology.

### 1.1. Economics Motivations

In a competitive sector such as the automotive industry, forging processes are widely used for the manufacture of automotive components working under load, such as parts for gearboxes, chassis, engines or transmissions. Forging represents the preferable option to obtain parts with complex geometry with appropriate mechanical properties and high productivity. Manufacturing of forging dies is quite expensive because of the high structural and usage demands, such as impact strength, mechanical wear and high temperature resistances. They are usually made of tool or high alloy steel.

Two thirds of total manufacturing costs are due to machining and polishing operations and are influenced by several factors; among them, the cutting tool is one of the elements that contribute to the cost of the final products [21]. Furthermore, dies quality depends largely on the state of wear of the cutting tool, therefore the importance of optimizing cutting tools wear during machining.

### 1.2. Scientific Motivations

Dies machined surface generally has a complex shape. These free forms are commonly machined by ball end nose cutting tools as the cutter readily adapts well to the curvatures [22]. However, the tip of these tools has a negative effect on performance machining, due to the zero value of cutting speed at this point [23,24]. 

During the cutting process the tool suffers mechanical stress, high temperatures and the corrosive effect of the refrigerant in case of use. The combination of these factors determines the tool life, either due to the progressive wear that occurs in areas of contact tool–chip or workpiece–tool or by the sudden fracture of the tool. Mechanical or thermal failures may occur due to improper use of cutting parameters or malfunction of the machine. When this breakdown occurs immediately the tool is useless. Unlike the sudden fracture, progressive wear is inevitable but can be controlled and predicted. 

**Figure 2:**
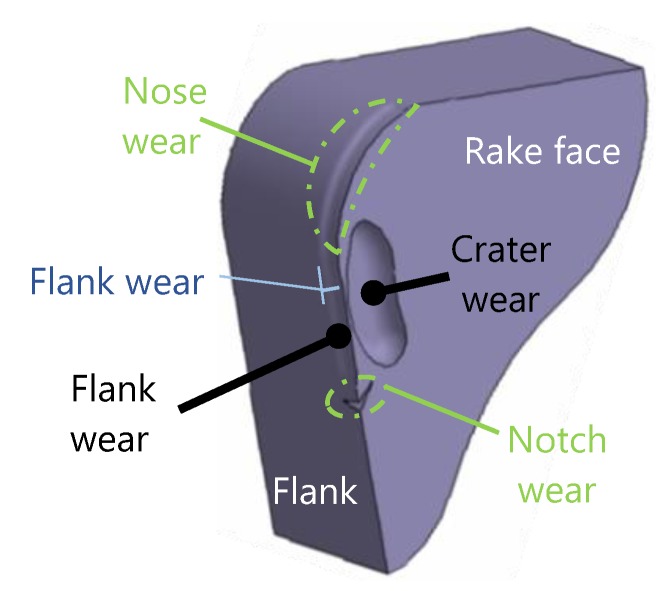
Principal locations and types of wear.

There are several types of tool wear caused by different mechanisms, the most common being abrasion, adhesion or oxidation [25]. The different types of tool wear are localized mainly to two areas as showed in Figure 2, flank and rake face [26]. To measure the wear, some parameters have to be established, as flank wear (VB), depth of crater wear (KT), length of crater wear (KM) or retraction of the cutting edge (KS) [27,28].

The principal aim of this paper is the presentation of simple, fast and inexpensive methodology for the wear kinetics monitoring of cutting edges in complex kinematics situations. Milling cutting process has been selected, where inserts have well-defined cutting edges and the kinematic is more complex than simple turning. For abrasive high added value finishing process (honing, lapping, polishing, belt finishing, etc.), the tools description is more complex and ambiguous than for cutting tools. 

For the present article, milling process offering various contact mechanics situations of cutting edge and work-piece with complex kinematics is discussed. The multifaceted dies manufacturing imposes that complex kinematics and demands the high surfaces morphology requests. For these reasons, the specific geometry is designed and the effect of wear on cutting edge can be observed. Cf infra chapter 3.

## 2. Experimental Strategy

**Figure 3:**
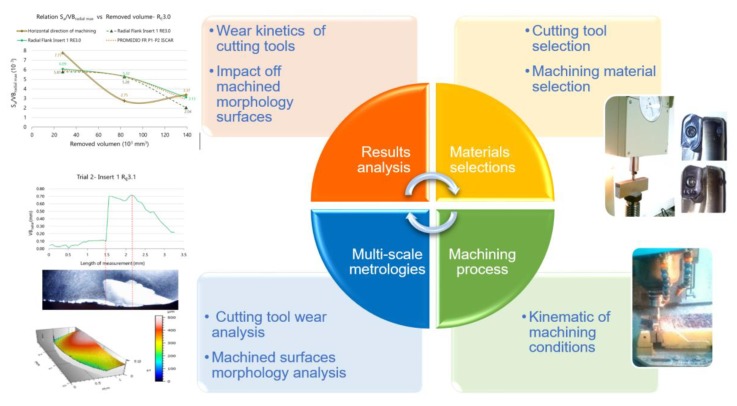
Experimental procedure.

The experimental design in present study is focused in die manufacture sector, and primarily applied at the finishing process of die cavities machining where very hard materials are machined. The main objective is to analyse the wear kinetic of cutting tools and how this wear rebound over the morphology of the machined surfaces. To accomplish these objectives, an experimental procedure, illustrated in Figure 3, was defined, where tool wear was measured and the topography of the machined surfaces was analysed to obtain the relationship between these parameters.

### 2.1. Workpiece Material and Geometry

One of the most widely materials used in the manufacture of forging dies is the AISI H13. It is a hot work tool steel, particularly a Cr–Mo–V alloyed steel (Table 1), and is characterized by good ductility, toughness, wear resistance, hardenability and machinability.

The material machined in this investigation undergo a heat treatment process of vacuum quenching and tempering. The heat treatment increases the hardness of the steel from 21 to 47 HRC (Table 2).

**Figure 4:**
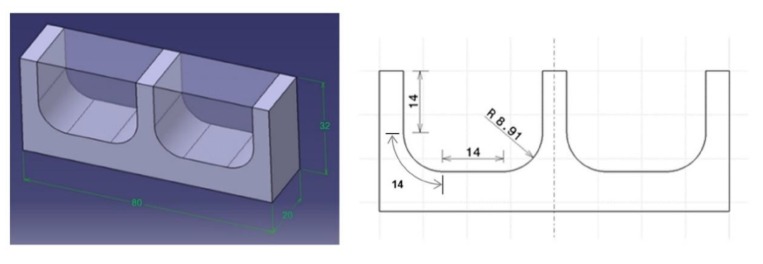
Final geometry of machined workpiece in AISI H13 material of dimensions 20 mm × 32 mm × 80 mm as it is shown.

The dies of high added value can have some convoluted cavities and complex geometries that tools have to follow during machining. After specific morphological studies in relation to dynamic of contact in die–tool interface, specific geometries can be experimentally explored. Ball-end milling tools are usually used in the machining of free-form surfaces in dies and the working tool diameter is constantly changing and, consequently, cutting conditions also change. Some researchers have studied the influence of surface inclination angle. Wojciechowski concluded that edge forces during ball end milling of hardness steel are depended on tribological phenomena in the word piece-tool flank interface, but the study for tool wear was carried out for a unique angle of 45° [29]. Miko conducts the study between 0° and 90° of inclination to study the working diameter of a ball-end milling tool during the machining, but the tool wear is not analysed [30]. In order to simulate the finishing operation of a die, in this study two cavities were selected in AISI H13 material of dimensions 20 mm × 32 mm × 80 mm as it is shown in Figure 4.

### 2.2. Tools and Machining Procedure

**Figure 5:**
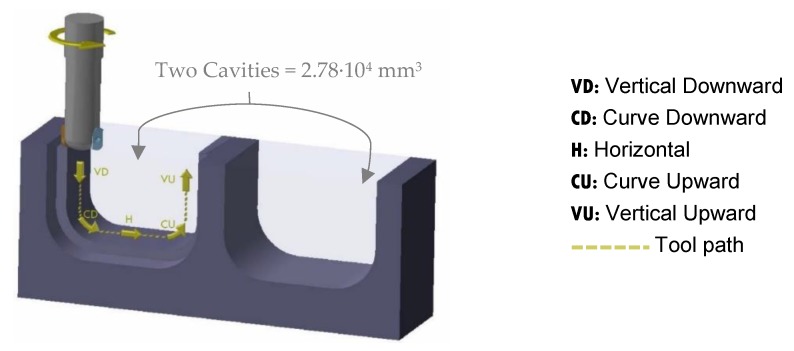
Machining kinematics.

The cavities were designed in order to describe five machining directions that a tool can follow during the machining of die cavities: vertical downward (VD), curve downward (CD), horizontal (H), curve upward (CU) and vertical upward (VU), represented in Figure 5. This geometry enforce that the length traced by the tool is the same in each direction with the defined machining strategy, having a final length of 14 mm in the last finish pass in each direction of each cavity. The removal volume has been calculated and corresponds to 2.78 × 10^4^ mm^3^ per cycle. Each cycle corresponds to machining a specimen with two cavities.

The strategy and tool path have been programmed using the CAM module of Catia V5^®^, following a strategy Z-Offset Sweep Roughing with one way tool path style in the machining of the two cavities in order to optimize the material in the experiments, with a machining tolerance, which allows a maximum distance between the theoretical and computed tool path, of 0.1 mm.

With the aim of reducing the negative effect of the tip of ball end nose tools during the machining, in this study, a flat end tool composed by two inserts of large nose radius was used for the finishing operations. The large nose radius allows for following during the study the complex geometry of cavities.

Milling experiments were performed in dry conditions with the machining parameters showed in Table 3, similarly to the industrial process in the forging industry company CIE Galfor. The cutting process has made in an Anayak Anak-Matic 7 milling centre that uses a Fanuc Numerical Controller.

**Figure 6:**
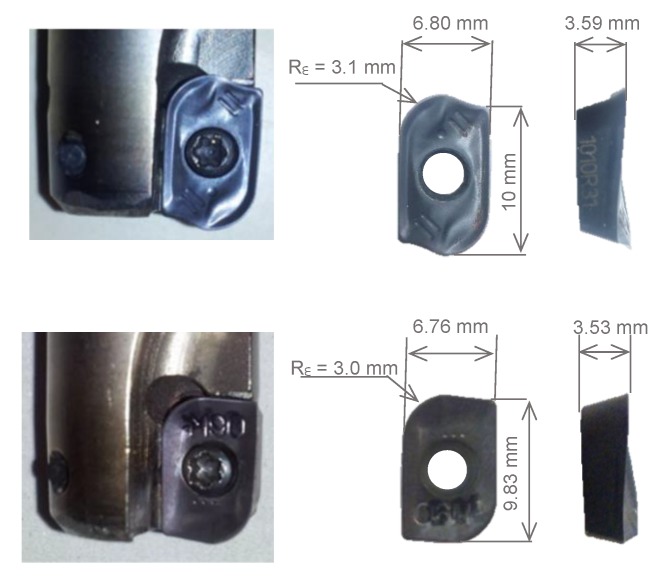
Commercial inserts selected for experimental investigations.

The tool has a diameter of 16 mm with two inserts. Figure 6 shows the two equivalent commercial inserts selected, corresponding to ISO APKT 11T3, that have been used in order to verify the methodology of measurements and also to draw conclusions about this methodology. Both inserts correspond to PVD-(Ti,Al)N coated cemented carbide tool with P10 classification, the first one has a nose radius of 3.1 mm (R_ε_3.1) and for the second one the nose radius is 3.0 mm (R_ε_3.0).

### 2.3. Experimental Approach

To set out a relationship between input (type of commercial insert and total removed volume) and output (tool wear and surface morphology), in the current study two equivalent commercial inserts with different tool nose radius (R_ε_3.1 and R_ε_3.0) have been used, and each of them remove three levels of volume (one, three and five specimens). Following an One-Variable-At-a-Time (OVAT) approach [31], six experimental trials were conducted as outlined in Table 4.

**Figure 7:**
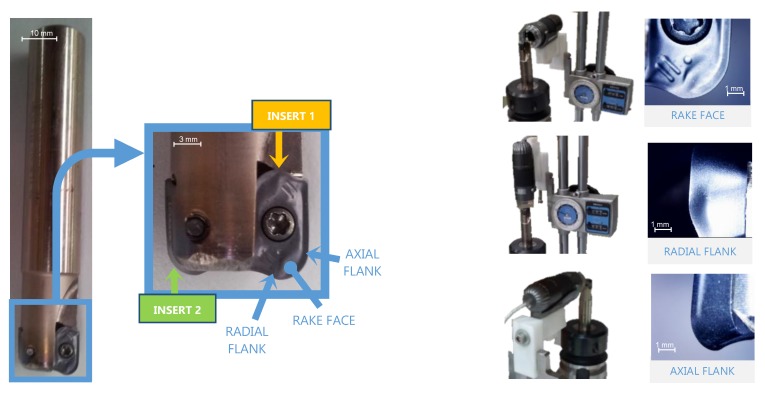
Optical images obtained of the inserts and microscope positioning to take inserts pictures.

After the machining of each specimen, optical images of both inserts of the tool were obtained with a digital microscope, as shown in Figure 7. Two images were obtained to check the flank wear (VB) corresponding to axial and radial flanks images, whereas the rake face images were obtained to measure retract of the cutting edge (KS). The images have been processed with In-Sight Explorer software from Cognex^®^.

**Figure 8:**
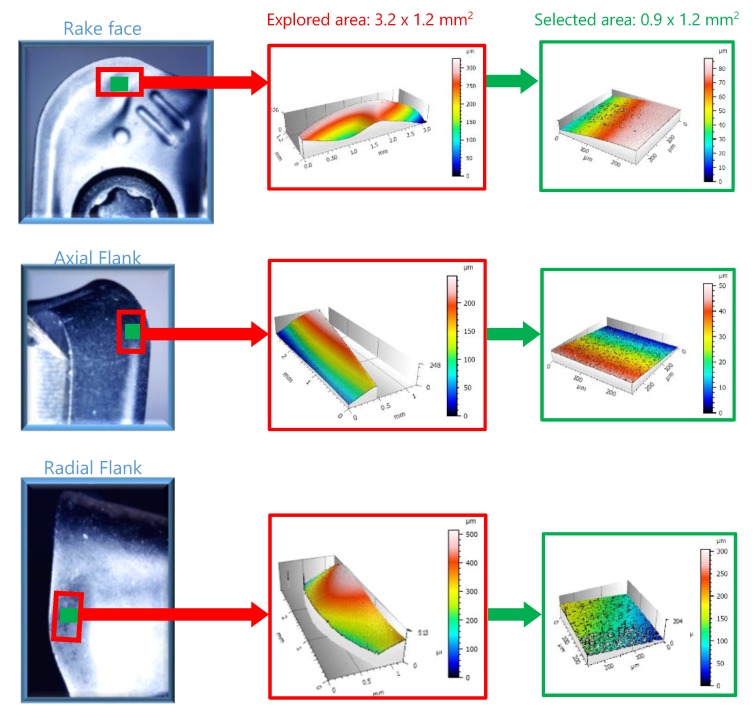
Areas of 3D surface measurement on inserts.

**Figure 9:**
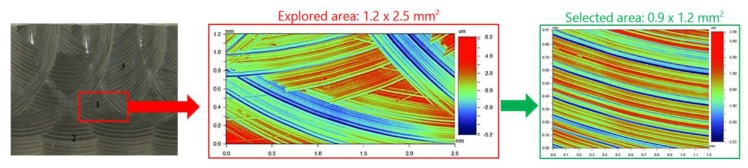
Areas of 3D surface measurement on horizontal machined surfaces.

At the end of each experimental trial, both, inserts and specimens, were analysed by the non-contact optical profiling system Wyko NT110, taken measurement arrays of 0.9 mm × 1.2 mm and a stitching large area on each area. On the inserts three areas were analysed by interferometry, corresponding with the areas of maximum wear observed in each optical image taken by the digital microscope (Figure 8). In the case of specimens, the analysis was realised in each machining direction. Figure 9 shows the analysis realised in the surface machined in horizontal direction.

### 2.4. Wear Metrological Algorithm

**Figure 10:**
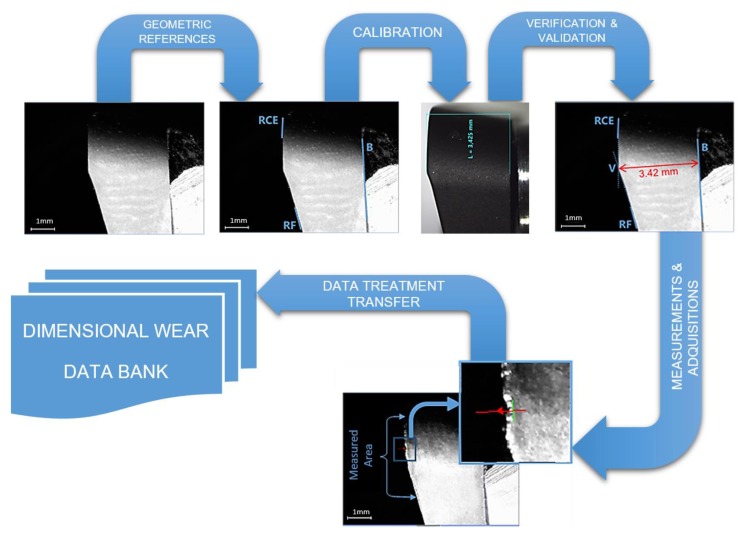
Wear metrological algorithm.

The usual procedure of Digital Image Analysis can be divided into the following steps: image acquisition, image processing, image analysis, recognition and interpretation [32]. In our study, optical images acquired by digital microscope have been treated to get the values of tool wear following the next steps (Figure 10).

- Filtering: Different filters proposed by Insight Explorer from Cognex^®^ were studied to get better results from the images. After the analysis, the results obtained did not improve the quality of tool wear measurements and the final analysis was applied without filtering.
- Reference: To locate the insert on the image, the invariable items have to be founded. These items are different in each kind of image (radial flank, axial flank and rake face).
- Calibration: To convert pixel measurements performed on the image to their corresponding values in the real world throughout some known values. Dimensions measured by a stereoscopic microscope were used as reference for calibration.
- Measurement: The pixels of the wear region were obtained on the calibrated image throughout the analysis along the tool border.

In the axial flank image a faint wear was appreciated, so the axial flank wear was not measured.

### 2.5. Radial Flank Wear Measurement (VB_radial_)

**Figure 11:**
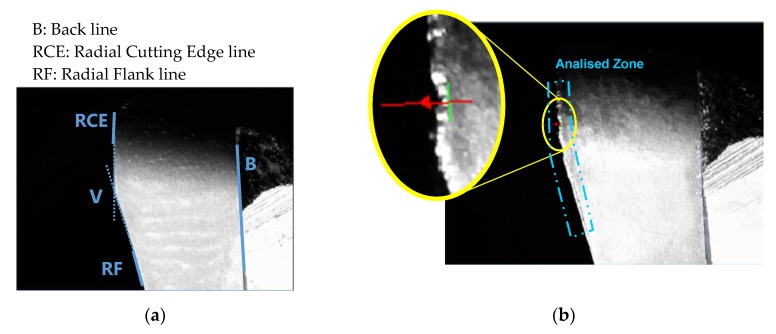
(**a**) References to radial flank images. (**b**) Zone of tool wear measurement.

To reference the images of radial flank, the lines B, RCE and RF were identified on the image as can be observed in Figure 11, taking into account that wear is produced around the point V, and then RCE and RF have to be identified in short lengths: RCE on the top of the insert and RF on the bottom. These lines are a one-dimensional projection of the image region by summing pixel values on radial line segments scanned in the positive y-direction relative to the region’s local coordinate system.

To calibrate the images, inserts were measured with a stereoscopic microscope, obtaining the distance between the point V and the line B. 

Once images were calibrated, the wear was measured along a distance on the border of the radial flank instead of quantification the maximum wear VBmax on the insert [33]. 

Each image was treated as a grid of pixels. To perform wear measurement, pixels in a range along the tool cutting edge were identified on the images from the new insert (reference image) to the worn one, post machining insert after the last specimen of the cycle. 

**Figure 12:**
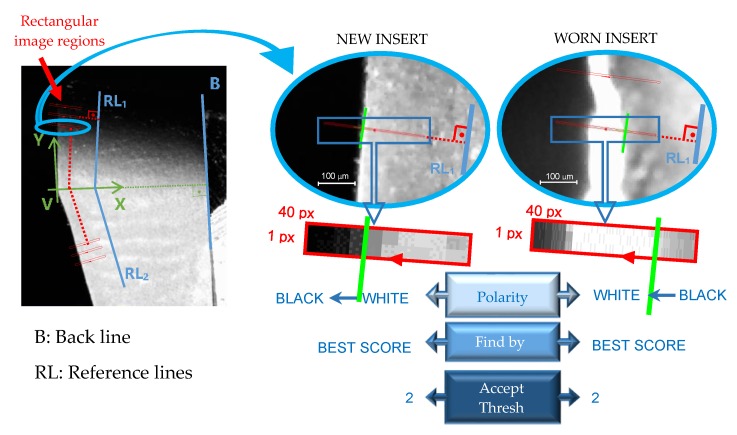
Pixels identification of the border on the tool cutting edge on radial flank images.

To measure wear in the perpendicular direction of the cutting edge, two reference lines were created parallel to the edges of the tool, taking a reference line above the point V (RL1 parallel to RCE) and another one for the bottom (RL2 parallel to RF), as can be seen in Figure 12.

In this figure it can be seen as the images are swept with rectangular image regions every 1 pixel-offset in the Y-axis direction to locate the pixels on the cutting edge. This location was performed to a pixels’ polarity with a minimum acceptable edge contrast of 2 in the grayscale histogram of the scan region, and by selecting the single edge with the best score technique. For the new insert images the polarity of the edge was established for white-to-black and for the worn insert images it was black-to-white scanning from right to left. 

The distance between each located pixel to the reference lines (RL_i_) were calculated and named as D_i_ (i = 0, 1, …, 5), where i represents the i-cycle of machining (number of specimens machined, being i = 0 the new insert).

### 2.6. Retraction of the Cutting Edge Measurement (KS)

**Figure 13:**
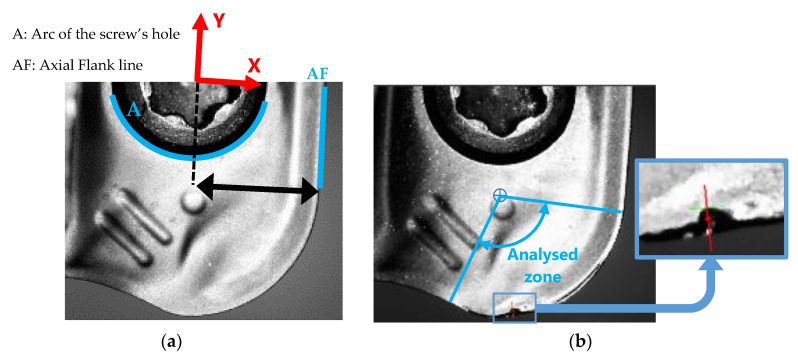
(**a**) References to rake face images. (**b**) Zone of tool wear measurement.

The reference in the rake face images is made by the diameter of the screw’s hole and the border of the insert. The arc A and the line AF were identified, as showed in Figure 13a. In the same way as radial flank images, the calibration was performed using the measurements taken with a stereoscopic microscope of the diameter of the screw’s hole and the insert width, obtaining then the distance between this hole centre and the line AF. 

The wear was measured along the tool nose radius (Figure 13b), similarly to VB_radial_ measurements, comparing pixels of the worn insert’s images with the ones of the new insert’s images. 

**Figure 14:**
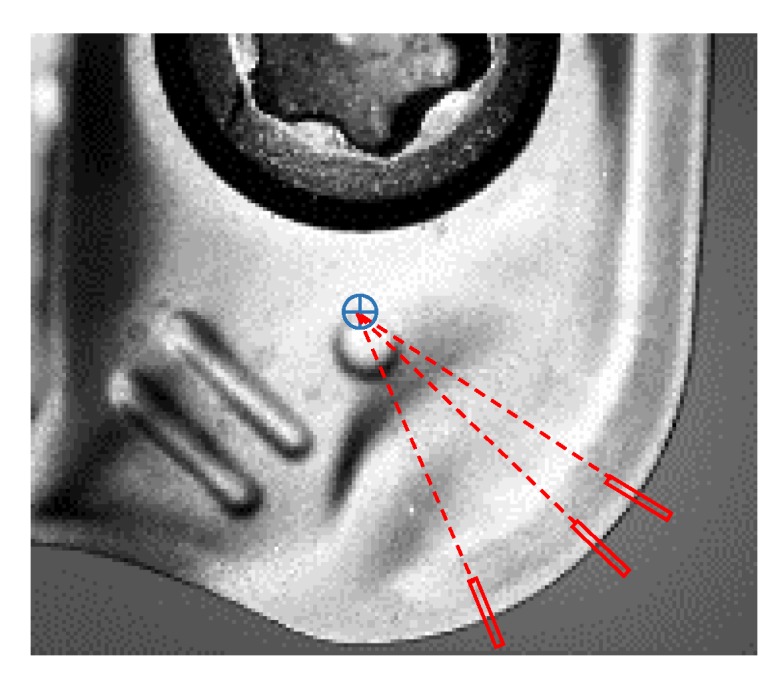
Pixels identification of the border on the tool nose edge on rake face images.

To measure wear in the perpendicular direction of the nose edge, pixels were found in the radial direction from the centre of the nose radius, identified it in the image of the new insert. These images were swept with rectangular image regions every 1 pixel-offset in the radius direction to locate the pixels on the nose edge, as showed in Figure 14. The same polarity parameters as in the radial flank images analysis were used, as was the best score technique to select the single edge. 

The radius in each pixel was calculated and named as R_i_ (I = 0, 1, …, 5), where i represents the i-cycle of machining (number of specimens machined, being i = 0 the new insert). 

## 3. Results and Discussion

### 3.1. Wear Kinetics of Radial Flank

**Figure 15:**
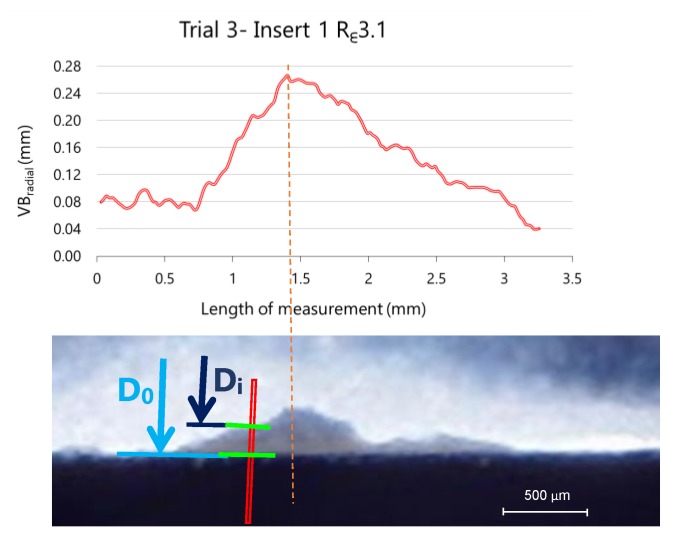
Measured VB_radial_ of insert 2 during trial 3 (removed volume 13.9 × 10^4^ mm^3^) and two overlapping images of radial flank: reference insert (background) and worn insert (first plane).

The tool wear at each pixel was calculated as the difference between these distances:

VB_radial_ = D_0_ − D_i_, (1)

where the distances D_0_ and D_i_ correspond to distances measured in the reference insert and the worn inserts, respectively, as shown in Figure 15. In this figure are also represented two overlapped images corresponding to the new insert (background image) and a worn insert, in this case the R_ε_3.1 insert after five cycles of machining (overlapped image).

Following the procedure described before, 48 radial flank images corresponding to the 6 experimental trials were analysed. On each image a length of 160 pixels along the cutting edge was studied. 

**Figure 16:**
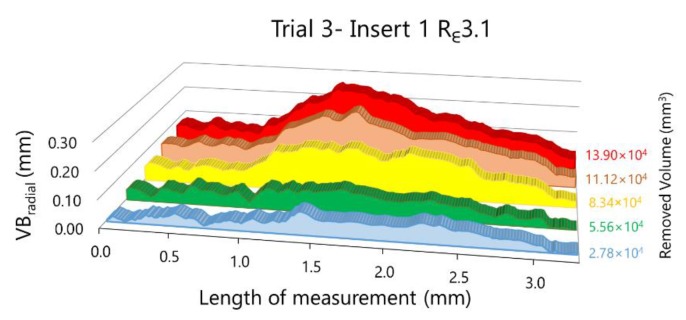
Evolution of VB_radial._

The results of the analysis for insert number 1 during trial 3 are showed in Figure 16, where six images were analysed (one of the new insert and five of the same insert after each cycle of machining, with a total of five cycles).

To smooth out punctual errors or fluctuations about pixel identification, a moving average with interval 4 is applied. This correction allows obtaining the wear evolution along the cutting edge, as can be observed in Figure 16. The difficulty of discrimination as well as surface morphology analysis offer supplementary tasks and information on wear process.

### 3.2. Wear Kinetic of Rake Face

The retraction of the cutting edge at each pixel was calculated as the difference between these distances

**Figure 17:**
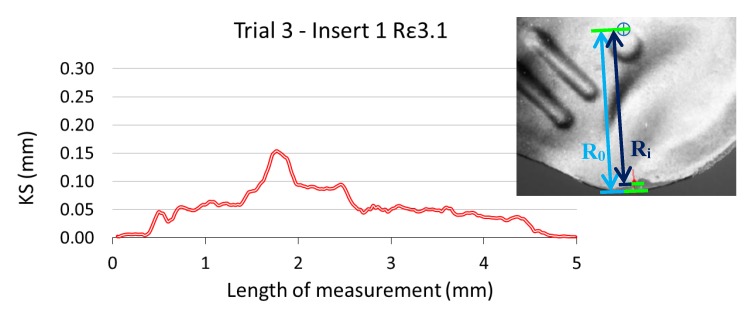
Measured KS of insert 1 during trial 3 (removed volume 13.9·10^4^ mm^3^) and two overlapping images of rake face: reference insert (background) and worn insert (first plane).

KS = R_0_ − R_i_, (2)

where the distances R_0_ and R_i_ correspond to radius measured in the reference insert and the worn inserts, respectively, as shown in Figure 17. Two overlapped images corresponding to the new insert (background image) and a worn insert (overlapped image) are shown in this Figure.

Following the procedure described before, and similarly to the radial flank analysis, 48 rake face images corresponding to the six experimental trials were analysed. On each image a length of 197 pixels along the cutting edge was studied. 

**Figure 18:**
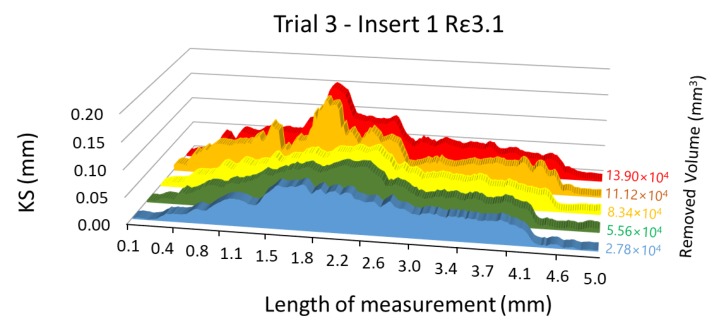
Evolution of KS.

The results of the analysis, applying a moving average with interval 4, for insert number 1 during trial 3 are showed in Figure 18, where also six images were analysed (one of the new insert and five of the same insert after each cycle of machining, with a total of five cycles).

### 3.3. Discriminatory Analysis of Two Similar Inserts

**Figure 19:**
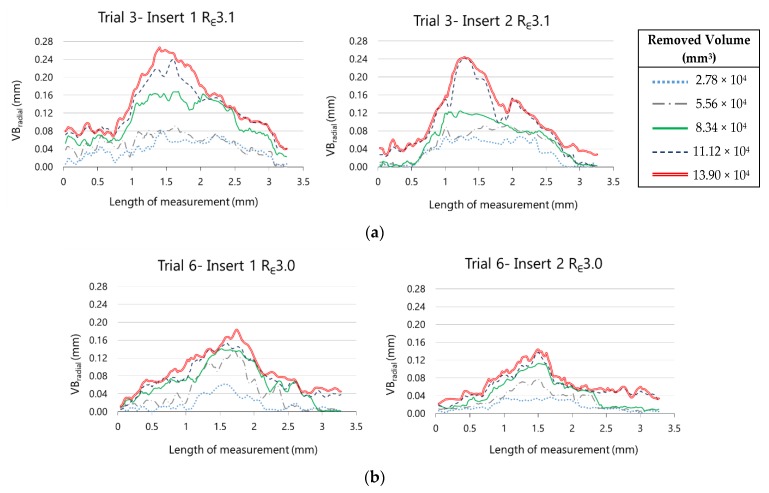
Evolution of the radial flank along the cutting edge on (**a**) R_ε_3.1 inserts and (**b**) R_ε_3.0 inserts.

In Figure 19, the evolution of the radial flank wear along the cutting edge for the two inserts as much from R_ε_3.1 as from R_ε_3.0 can be observed. These representations were taken from experimental trials 3 and 6, corresponding to machining hardened AISI H13 during five cycles with R_ε_3.1 and R_ε_3.0 tools, respectively.

In both experiments, the behaviour of VB_radial_ with R_ε_3.1 and R_ε_3.0 is similar between inserts 1 and 2. The maximum value of VB_radial_ for insert 1 is bigger than for insert 2, as much for R_ε_3.1 as for R_ε_3.1. This representation allows us not only to compare the maximum value of both inserts in a tool but also to compare the extension of the wear along the cutting edge. We can observe also the evolution of radial flank wear along the cutting edge to the extent that material is removed. 

**Figure 20:**
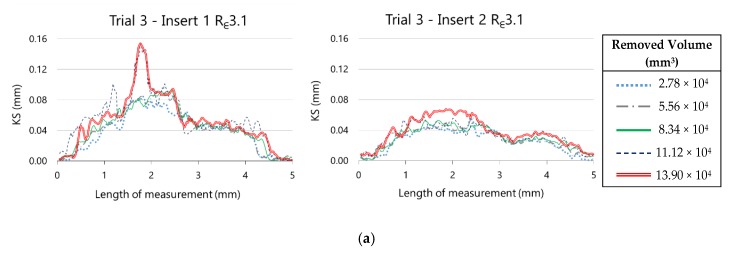

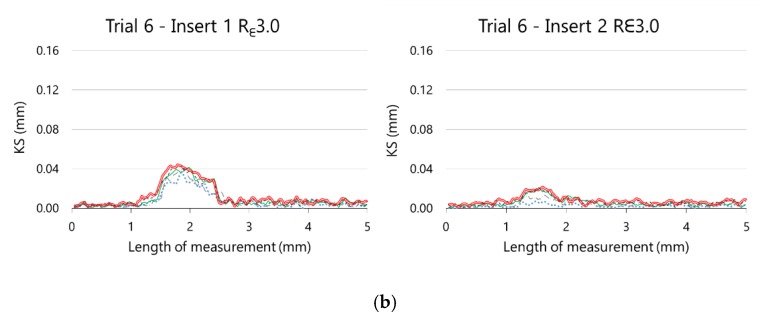
Evolution of the retraction of the cutting edge along the nose edge on (**a**) R_ε_3.1 inserts and (**b**) R_ε_3.0 inserts.

Similar conclusions are drawn from the analysis of the evolution of retraction of the cutting edge, showed in Figure 20.

**Figure 21:**
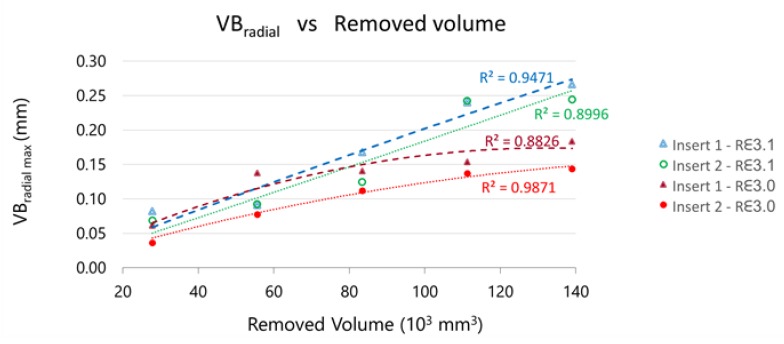
Evolution of maximum VB_radial_ versus the removed volume during machining.

The maximum value of VB_radial_ can be extracted for each insert from the previous figures. On Figure 21, the evolution VB_radial_max_ with the removed volume is represented.

The initial VB_radial_max_ is similar for both tools (R_ε_3.1 y R_ε_3.0), but the R_ε_3.1 tools wear quickly than the R_ε_3.0 ones during the machining, as can be observed in the figure.

### 3.4. Discriminatory Detection of Catastrofic Wear

**Figure 22:**
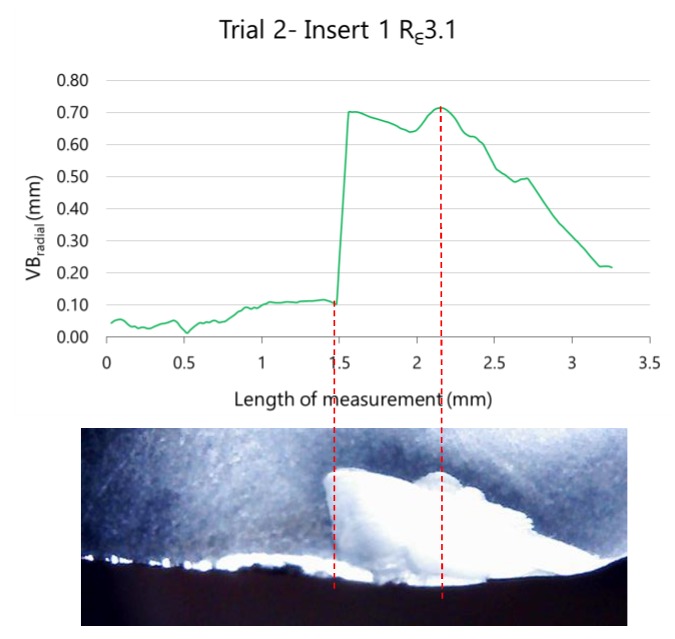
Fracture of insert 1 during trial 2 (removed volume 8.34 × 10^4^ mm^3^) with R_ε_3.1 tools.

Besides the analysis of VB_radial_max_ and the length of wear along the cutting edge, this methodology of image analysis allows the detection of fractures or chip rests on the insert that could not be detected if only the maximum wear is studied. An example is shown in Figure 22. Robustness of algorithms involves the precision of contrast analysis.

## 4. Conclusions and Perspectives

As it has been demonstrated (cf supra Chapter 3), cheap and simple non-contact methodology is possible to be implemented on machining process to follow reasonably wear process of cutting tools. The experiments carried out in the measurement of the images have provided valid results for the definition of the state of the tool. The robustness of algorithm has been corroborated by checking the results with wear measurements of the inserts in Stereoscopic Zoom Microscope SMZ800.

To measure tool wear by optical systems, measurement along the entire flank is more accurate than obtaining the maximum wear value. In addition, this measurement procedure allows to establish different areas of study of wear depending on process parameters such as the machining direction or the depth of cut. The use of the tool could be further optimized based on these parameters and the more worn areas.

The methodology described in detail is robust and easy to be applied on manufacturing cutting machining. Wear kinetics can be established and self-tuning adaptive control can be envisaged.

## Figures and Tables

**Table 1:** Typical chemical analysis (%) of machined material.

C	Si	Mn	Cr	Mo	V
0.39	1	0.4	5.2	1.4	0.9

**Table 2:** Rheological properties of machined material.

Hardness	Tensile Strength Rm	Yield Strength Rp0.2
47 HRC	1420 MPa	1280 MPa

**Table 3:** Machining parameters.

Machining Parameter	Value
Tool path style	Monodirectional
Machining tolerance	0.01 mm
Radial depth a_p_	3 mm
Axial depth a_e_	0.25 mm
Speed v_c_	120 m/min
Feed rate f	0.24 mm/rev

**Table 4:** Design of experiments.

Trial	Tool Nose Radius of Insert	Total Removed Volume mm^3^	Machined Specimens
1	R_ε_3.1	2.78 × 10^4^	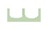
2	R_ε_3.1	8.34 × 10^4^	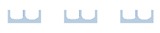
3	R_ε_3.1	13.9 × 10^4^	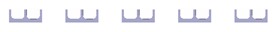
4	R_ε_3.0	2.78 × 10^4^	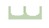
5	R_ε_3.0	8.34 × 10^4^	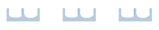
6	R_ε_3.0	13.9 × 10^4^